# Dropout or Drop-In Experiences in an Internet-Delivered Intervention to Prevent Depression and Enhance Subjective Well-Being During the Perinatal Period: Qualitative Study

**DOI:** 10.2196/46982

**Published:** 2023-12-22

**Authors:** Lisbeth Valla, Silje Marie Haga, Susan Garthus-Niegel, Filip Drozd

**Affiliations:** 1 Regional Centre for Child and Adolescent Mental Health, Eastern and Southern Norway, Oslo, Norway; 2 Department of Nursing and Health Promotion, Faculty of Health Sciences, Oslo Metropolitan University, Oslo, Norway; 3 Institute and Policlinic of Occupational and Social Medicine, Faculty of Medicine, Technische Universität Dresden, Dresden, Germany; 4 Department of Childhood and Families, Norwegian Institute of Public Health, Oslo, Norway; 5 Institute for Systems Medicine and Faculty of Medicine, Medical School Hamburg, Hamburg, Germany

**Keywords:** perinatal depression, internet intervention, dropout, well-being, perinatal period

## Abstract

**Background:**

The perinatal period is a vulnerable time when women are at increased risk of depression. “Mamma Mia” is a universal preventive internet-delivered intervention offered to pregnant women, with the primary goals of preventing the onset or worsening of depression and enhancing subjective well-being during the perinatal period. However, treatment dropout from internet-delivered interventions is often reported.

**Objective:**

The study aim was to acquire an understanding of the different experiences among participants who dropped out of the Mamma Mia intervention during pregnancy, compared to participants who dropped out during the postpartum follow-up phase.

**Methods:**

A total of 16 women from a larger randomized controlled trial (Mamma Mia) participated in individual semistructured interviews following a strengths, weaknesses, opportunities, and threats format. Of the 16 participants included, 8 (50%) women dropped out early from the intervention during pregnancy (pregnancy group), whereas 8 (50%) women dropped out later, after giving birth (postpartum follow-up group). Data were analyzed using the framework approach.

**Results:**

The results showed that there were differences between the groups. In general, more participants in the postpartum follow-up group reported that the program was user-friendly. They became more aware of their own thoughts and feelings and perceived that the program had provided them with more new knowledge and practical information than participants in the pregnancy group. Participants in both groups suggested several opportunities for improving the program.

**Conclusions:**

There were differences between women who dropped out of the intervention during pregnancy and the postpartum follow-up phase. The reported differences between groups should be further examined.

## Introduction

Mental health problems are one of the leading causes of disability worldwide. In particular, depression constitutes a major public health problem [1]. Women are particularly susceptible to depression throughout the perinatal period [2]. During this period, between 10% to 15% of women experience moderate to severe depressive symptoms [3,4]. Depression is commonly associated with a range of negative consequences for the woman (eg, reduced life quality and social functioning); it may also affect the mother-infant relationship and have long-term consequences for their child [5-10] and partner [11,12].

Despite the availability of different types of traditional treatments for mental health problems, help-seeking behavior among women with perinatal depression remains low (ie, 17%-25% [13-15]). The negative consequences of maternal depression for the child and the whole family highlight the importance of adequate and timely treatment [8]. Internet-delivered interventions have emerged as an innovative approach to prevent and treat depression. Recent studies have demonstrated their usability, feasibility [16-18], and effectiveness in reducing depressive symptoms [19-24]. However, treatment dropout is often reported, both in unguided and guided internet-delivered interventions [25,26]. Dropout is often preceded by missed sessions or ending without completing the treatment components [27]. Research considers a lower treatment completion rate as a moderator of treatment effect size [28]. It is important to understand participants’ reasons for dropout. Exploring and comparing dropout at different stages helps researchers identify and understand the underlying causes of dropout at different stages of intervention. Researchers have previously found that baseline symptoms of depression, depression with comorbid anxiety symptoms, lower educational levels, interventions without guidance, and gender are risk factors for dropout from psychological web-based interventions for depression [29-31]. Dropout is also frequently cited as being caused by the perception of lengthy and time-consuming content, social changes, the demands of caring for a newborn, a lack of motivation to begin the intervention, engagement with the content and relationship with technology, and feelings of benefits from the intervention [26,32,33].

A qualitative systematic review and meta-synthesis across 24 quality studies was aimed at exploring the views of people who had been invited to participate in digital health interventions for depression [34]. Three themes emerged regarding acceptability and usability: initial motivations and approaches to digital health interventions, the personalization of treatment, and the value of receiving personal support in digital health interventions. The meta-synthesis suggests that participants’ initial beliefs about digital health interventions can have an important effect on their engagement with these types of interventions.

In this study, we focused on an internet-delivered, unguided self-help program for depression (“Mamma Mia”), to achieve a deeper understanding of user experiences. Mamma Mia is designed as a universal preventive intervention that can be offered to all pregnant women. Its’ primary goals are to prevent the onset or development of depression and enhance subjective well-being during the prenatal and postnatal periods. Examining participants’ experiences from a qualitative perspective can provide more in-depth answers about the complexity of treatment dropout. The study aim was thus to acquire an understanding of the different experiences among participants who dropped out of the intervention during pregnancy, compared to participants who dropped out during the postpartum follow-up phase.

## Methods

### Study Design and Participants

This study used a qualitative design with individual semistructured interviews following a strengths, weaknesses, opportunities, and threats (SWOT) format. Participants were recruited through the Mamma Mia randomized controlled trial [20,35], where pregnant women in Norway were invited to participate between December 2013 and February 2015. They were recruited at well-baby clinics, during routine prenatal care, and via hospitals in Eastern Norway during regular ultrasound imaging (gestational wk 18-20). Eligible participants had to be pregnant (up to gestational wk 25), 18 years or older, able to read and write Norwegian, and have access to the internet and an email account. All participants were enrolled for the study, enrolled in the intervention, and invited for an interview, consecutively. Inclusion criteria for being invited to an interview were either (1) completing the intervention, (2) having no program activity during the last 4 weeks, or (3) lagging 3 or more sessions behind the prescribed intervention schedule. Participants fulfilling the 2 latter criteria were defined as dropouts.

In this study, 16 interviews were conducted. Respondents were women who were either pregnant or had given birth at the time of the interview. Of the 16 participants included, 8 (50%) women dropped out early in the intervention during pregnancy (pregnancy group), whereas 8 (50%) women dropped out later, after giving birth (postpartum follow-up group). Participants in the pregnancy group had completed between 3 to 15 sessions out of a total of 44 sessions. At the time they were invited to interview, these 8 women had no program activity during the last 4 weeks or were lagging 3 or more sessions behind the prescribed intervention schedule. They had left the program before it ended at session 44 (after birth). Participants in the postportum follow-up group had completed between 25 to 38 sessions out of a total of 44 sessions. They had followed the intervention from session 1 (during pregnancy) to sessions 25-38 (after birth); however, they did not complete the intervention, and at the time they were invited to the interview, they had had no program activity during the last 4 weeks or were lagging 3 or more sessions behind the prescribed intervention schedule.

### Mamma Mia

Mamma Mia is a universal internet-delivered intervention developed with the primary goals of improving or maintaining subjective well-being and preventing the onset of or reducing depressive symptoms during pregnancy up to 6 months after birth. Overall, Mamma Mia consists of 44 sessions over 11 months in 3 phases, starting from pregnancy between gestational weeks 17 and 24 and lasting into the postpartum period 6 months after childbirth. The first phase, the pregnancy phase, consists of 16 sessions, which starts at gestational week 21 and ends at week 40. The second phase is the maternity phase, which starts when the infant is 2-3 weeks old and lasts for 6 weeks. Sessions are delivered 3 times a week, for a total of 18 sessions. The final phase is the low-intensity follow-up phase, consisting of 10 sessions over 18 weeks. These sessions are delivered with some variation (weekly at first and then biweekly). All sessions include themes specific to the perinatal period. Mamma Mia was deployed as an unguided, universal intervention with a tunneled design to guide women through the program in a step-by-step fashion, in accordance with the psychological preparation of becoming a mother. Each session was designed to take 10-15 minutes. Mamma Mia has a website that could be use on tablets and mobile devices, and the intervention is delivered through email and interactive websites that include text, pictures, prerecorded audio files, and user feedback [36]. In the Mamma Mia randomized controlled trial, the total number of respondents in the total sample was 1342 at baseline; 1117 (83.2%) at gestation week 37; and 962 (71.7%), 886 (66%), 847 (63.1%) at 6 weeks, 3 months, and 6 months post partum, respectively.

### Interviews

The semistructured interviews followed the theory-neutral SWOT framework, focusing on participants’ spontaneous and open appraisals of Mamma Mia. Each participant was asked questions about what they perceived to be the SWOT to Mamma Mia. During the first part of the interview, participants spoke freely about the different SWOT. In the second part of the interview, a more exploratory approach was used to obtain additional information and a richer description of the SWOT described in the first part of the interview. The SWOT format can be helpful to understand the intervention better and provide potentially more accurate perspectives on the intervention.

The interviews were conducted from March 2014 to April 2015 and were carried out at the Regional Centre for Child and Adolescent Mental Health. All interviews were recorded electronically and transcribed verbatim. The length of the interviews ranged from 48 to 110 (mean 75.8) minutes.

### Analysis

Descriptive statistics and frequencies were used to describe the characteristics of the sample. Interviews were analyzed using the framework approach as suggested by Gale et al [37]. The framework approach consisted of 5 phases: familiarization, identification of a thematic framework, indexing, charting, and mapping and interpretation. Familiarization involved immersion in the data by reading the transcripts several times. In developing the thematic framework, we identified themes from issues that the participants raised themselves. Indexing involved systematically applying the thematic framework to all interview transcripts. During the charting phase, we lifted data from their original context and rearranged them according to theme. We identified themes and patterns within and between the 2 different groups of participants: the pregnancy group and the postpartum follow-up group. In the mapping and interpretation phase, we reviewed the charts, compared and contrasted the data, and sought patterns and explanations within the data. NVivo (version 12; Lumivero) was used to structure the condensed meaning units and group differences. The final interviews did not reveal new information or insights, and it was considered that data saturation had been reached. To contribute to reflexivity in the research, continuous reflection was applied throughout the research processes on the researcher’s role, biases, values, and relationships.

Frequency labels were used to characterize the data. The “general” label corresponds to all or all but 1 case (n=7-8); the “typical” label corresponds to more than half of the cases up to the cutoff point for the “general” label (n=5-6); and the “some” label corresponds to 2 cases up to the cutoff point for the “typical” label (n=2-4). When comparing the 2 groups, we defined the results as different if they varied by at least 2 frequency labels [38].

### Ethical Considerations

The study has been performed in accordance with the Declaration of Helsinki and has been approved by an appropriate ethics committee, the Norwegian Regional Committees for Medical and Health Research Ethics (project REK-SØ 2012/1716). The study was conducted in line with the COREQ (Consolidated Criteria for Reporting Qualitative Research) checklist to promote transparency. Written informed consent was obtained from all participants upon recruitment. The Regional Centre for Child and Adolescent Mental Health, Eastern and Southern Norway, has granted permission to access and use the data set. The participants did not receive financial compensation.

## Results

### Participants

In the pregnancy group, the age ranged from 26 to 40 years, with a mean age of 31 years. One of the participants in this group was not a native Norwegian. In the postpartum follow-up group, the age ranged from 21 to 33 years, with a mean age of 29 years, and all the women were native Norwegians. In both groups, all women were married or cohabitating. They were highly educated—in both groups, 38% (3/8) had 1-3 years of college or university education, and 62% (5/8) had ≥4 years of college or university education—and all were currently employed. They had few symptoms of depression as measured by the Edinburgh Postnatal Depression Scale [39]; mean scores were 6.8 (SD 5.1) and 6.1 (SD 5.9) in the pregnancy and postpartum follow-up groups, respectively. At baseline in the pregnancy group, the mean Satisfaction With Life Scale [40] score was 21.1 (SD 3.2), mean positive affect score was 37.0 (SD 6.7), and mean negative affect score was 18.50 (SD 7.3). At baseline in the postpartum follow-up group, the mean Satisfaction With Life Scale score was 21.5 (SD 3.9), mean positive affect score was 31.8 (SD 3.06), and mean negative affect score was 22.25 (SD 10.12).

Table 1 shows the 4 primary themes that emerged from the interviews in the 2 groups: user-friendliness, awareness, learning, and adaption.

**Table 1.** Perceived experiences of the Mamma Mia intervention as reported by participants in the pregnancy and postpartum follow-up groups.

Themes		Pregnancy group	Postpartum follow-up group
**User-friendliness**			
	Similarities in subthemes	Easy to use Technical problems Time-consuming to follow up	Easy to use Technical problems Time-consuming to follow up
	Differences in subthemes	N/A^a^	Accessibility Simple design Weekly reminders Positive repetition of information Difficult to catch up
**Awareness**			
	Similarities in subthemes	Relationship to the partner	Relationship to the partner
	Differences in subthemes	Awareness of the pregnancy phases	Conscious of thoughts and feelings Connection to the baby Normalization of emotions Reflection
**Learning**			
	Similarities in subthemes	Evidence-based information	Evidence-based information
	Differences in subthemes	Instructive exercises	New knowledge New tools
**Adaptation**			
	Similarities in subthemes	Follow-up	Follow-up
	Differences subthemes	Adaptation to the target group Relevant information	Flexibility in the program More information or links to external sources

a N/A: not applicable.

### User-Friendliness

There was a group difference in the perceived user-friendliness of the program. Only some of the women in the pregnancy group mentioned that the program was easy to use, whereas women in the postpartum follow-up group generally reported that the program was user-friendly. When they reported that the program was user-friendly, they highlighted the accessibility of the program, the design, the weekly reminders, and the repetition of information to increase usability. They emphasized that the intervention contained brief, relevant information and appropriate reminders for the user. They also favored the program over other websites or web-based sources of information.

*It was very easy to log in, click on the links, and follow the program. It was also very easy to use...There was an okay amount of information*. [Respondent 238]

*I appreciate that the program is friendliness. You click on the E-mail, and you don’t need a password. It is an advantage with weekly reminders*. [Respondent 8]

*It is very easy to use. I have it on my phone, an email comes up. So, it is very user-friendly*. [Respondent 173]

At the same time, participants in both groups typically experienced technical challenges with the program. They reported that the program did not always work well on mobile phones and tablets. It was also difficult to store and retrieve information and videos in the program, especially when using a mobile phone. As 1 woman in the pregnancy group said: “The program did not work very well on my phone. It should be easier to follow the program if it had worked on my mobile” (respondent 327)

In general, participants in both groups also reported that the program was time-consuming with frequent emails and many program days. They did not always have time to complete the sessions according to the prescribed program schedule, especially if they had other children to care for. When they fell behind schedule, it felt difficult to catch up again, and for some women, this was perceived as stressful. A participant in the postpartum follow-up group said: “It’s challenging to spend so much time with your PC. Before I had the baby, it was nice to get an email and pay attention to the program. But after having the baby, I simply did not have the time” (respondent 8).

A participant from the pregnancy group said: “I used the program more actively in the beginning. I had decided to prioritize it” (respondent 248).

### Awareness

After participating in Mamma Mia, participants in the postpartum follow-up group generally reported that they had become more aware of their own thoughts and feelings than participants in the pregnancy group.

*I became more aware of my own thoughts, feelings, and moods...It made me aware of processes in myself*. [Respondent 238]

*I'm more aware of all these emotions, and became a little more...[have] gotten more in touch with myself*. [Respondent 279]

Some participants in the pregnancy group talked about awareness in relation to the phases of pregnancy and in their relationship with their partner. In contrast, women in the postpartum follow-up group typically talked about the changes that occurred during the transition to motherhood, as well as how the information in the program created awareness and self-reflection and contained important reminders that facilitated attachment and bonding with the baby. They emphasized that themes containing information about the baby were one of the strengths of the program.

*You feel like you are better able to connect with your baby. It strengthens the bond with the baby...and the connection is something that comes gradually*. [Respondent 155]

*I can now see how my baby responds to different things*. [Respondent 218]

The postpartum follow-up group also mentioned the ways in which the program helped them in their daily life by looking at things more positively. In addition to normalizing feelings, during pregnancy or in situations where it was difficult to comfort the child, they gained a greater understanding that they were not alone in experiencing such situations.

*It is useful to learn that people may have the same kind of challenges with the child as you. I became aware that some situations are normal, and I am not the only one who has these experiences*. [Respondent 202]

*Many people, my-self included, have concerns. When you are told that, for example, negative thoughts are normal, it feels good*. [Respondent 251]

### Learning

There was a difference between the groups in terms of learning outcomes. Learning was not to any great extent reported in the pregnancy group, whereas respondents in the postpartum follow-up group generally reported that the program had provided them with new knowledge and practical information that were useful. However, in the pregnancy group, relaxation exercises, such as mindfulness exercises, were perceived as pleasant and instructive by some respondents. Participants in the postpartum follow-up group typically reported that they had gained knowledge through information and interactivity in the program. They highlighted knowledge such as how to comfort the child, the stages of infant sleep-wake cycles, and how to manage conflicts in their relationship with their partner. This was useful because they used it in their interaction with their child and partner.

*There is a lot of practical information that you need as a new parent. I learned a few techniques, for example, to distinguish between the different stages of sleep. I learned a lot from that*. [Respondent 155]

*I learned a lot of things that I wasn't fully aware of, such as comforting the child*. [Respondent 279]

*I really think that the whole program was very educational*. [Respondent 238]

In general, participants in the postpartum follow-up group described that they changed their thoughts, feelings, and ways of interacting with their child and partner by acquiring new knowledge. They received practical strategies that influenced how they adapted to different requirements and environments, whether this applied to their child or partner. Participants said that they have been given new useful tools. For example, methods such as “gradual comforting” and managing infant sleep-wake cycles were mentioned as useful tools that were translated into practical strategies, helping them consider “what should be done” and “how it should be done.”

*It was very exciting...You don’t necessarily solve all problems, but you get some tools that can be used to solve problems constructively*. [Respondent 155]

*When a situation gets difficult, I now have a strategy to use*. [Respondent 238]

They appreciated that the content provided in the program was quality assured, in contrast to other information found on the web. They described the information that was given as trustworthy, and a couple of participants in both groups highlighted that the information in the program was evidence based. However, although the content was relevant, some of the participants also said that some exercises were difficult to implement. Difficult exercises that were mentioned were techniques intended to improve communication skills and facilitate conflict resolution with their partner.

### Adaptation

Participants in both groups suggested several opportunities for improving the program. Women in the pregnancy group typically suggested that the content of the program should be better adapted. They perceived that the content of the program was aimed at first-time mothers and suggested that the program should also be addressed to a greater extent to multiparous women. Some respondents also reported that some of the content in the program was familiar or not very relevant. Some sessions were considered too brief, and they emphasized the lack of depth and relevance of the information as central weaknesses. Furthermore, they suggested a parallel program dealing with problems concerning difficulties relevant to multiparous women.

*The challenge is that...there are people with different personalities and situations in one program. It is difficult to find a program that could be adapted to everyone*. [Respondent 248]

*The fact that I am a third-time mother may have affected me in the way I perceive Mamma Mia...It could have been divided into first- and second-time mothers, and asked questions about how the current pregnancy is different from the previous pregnancy*. [Respondent 32]

It was, in general, more pronounced in the postpartum follow-up group that they wanted more flexibility in the program. They reported dissatisfaction with the tunneled sequence—having to complete 1 module before starting the next one. A better adaptation of the program, where participants can drop some of the modules or reenter the program later, may help them perceive the program as being less stressful. The lack of flexibility was also mentioned with regard to subjects the women wanted to learn more about. Some sessions were considered too brief and superficial. Therefore, participants suggested links to web portals or other sources to retrieve more information of interest. The participants expressed a desire to go deeper into 1 subject of interest. Specifically, a link to more information about practical care for the infant and sleep was mentioned.

*It’s really about making suggestions about where to find more information, quality-assured information.* [Respondent 255]

*Access to resources, libraries or something where you have the opportunity to find out more*. [Respondent 155]

Some respondents in both groups, regardless of how long they had used the program for, wanted further follow-up when something was difficult.

…*that I get further help to figure out what to do next when I’m very depressed, or something is wrong. A description of how to proceed to get help*. [Respondent 8]

## Discussion

### Principal Findings

This study aimed to acquire an understanding of the participants’ experiences of a universal, internet-delivered intervention offered to all pregnant women, with the primary goals of preventing the onset or worsening of depression and enhancing subjective well-being during the perinatal period. We compared the experiences between participants who dropped out during the pregnancy phase to participants who dropped out during the postpartum follow-up phase. The analysis resulted in 4 themes, each relating to different experiences with the intervention. The results showed that there were similarities but also differences between the groups. The postpartum follow-up group had a larger proportion of participants who reported that the program was user-friendly. They also became more aware of their own thoughts and feelings and perceived that the program had provided them with more new knowledge and practical information than participants in the pregnancy group. However, respondents in both groups suggested several opportunities to improve the program. Although women in the pregnancy group typically suggested that the content of the program should be better adapted to the target group, it was generally more pronounced in the postpartum follow-up group that they wanted more flexibility.

Despite these findings, Mamma Mia was described as a positive and credible intervention in both groups. The main different between the group was that, in general, more women in the postpartum follow-up group reported that the program was user-friendly; emphasized the accessibility of the program; and indicated that the intervention containing brief, relevant information and reminders suitable for the user. They reported that the presentation and navigation in the program was easy to use and understand and was aesthetically pleasing, which was mainly due to the design. The experiences of user-friendliness may have contributed to the fact that women in the postpartum follow-up group followed the intervention longer than women in the pregnancy group. Users of internet-delivered interventions often experience barriers and difficulties when using new technology [26,41], but a design that is easy to use and perceived as user-friendly can reduce this burden [42]. It can thus contribute to maintaining engagement and achieving benefits from digital health interventions [43-45]. However, technical challenges with the program were mentioned by participants in both groups. They reported that the program did not always work well on mobile phones and tablets and that it sometimes was difficult to store and retrieve information and videos in the program, especially when using a mobile phone. This issue is important since previous research has shown that improving the convenience of internet programs improves usability and reduces attrition [26,41]. Our findings add to this evidence, indicating that there is still great potential for improvement in the design of eHealth products to provide technology that users will participate in and use.

Women in both groups also typically reported that the program was time-consuming and that they did not always have time to complete the sessions as prescribed according to the program schedule. Giving birth and caring for a new infant mark an important transition in life. Although this transition is often exciting and rewarding, it also leads to a multitude of new responsibilities, often alongside a range of physical, psychological, and social changes [46]; this may have made it difficult to follow the program on a weekly basis post partum. However, adaptation to a maternal role is affected by individual factors, such as educational background, their partner, and psychological state (eg, depression) [47-49]. The literature has previously documented the influence of external factors on treatment dropout [50,51], stating that the demands these factors place on the individual will lead to dropout if they are viewed as an obstacle to the individual’s daily life [51]. Lagging behind may result in perceiving the program as stressful, and the demands of motherhood may directly interfere with their ability to complete the program [52].

Differences between the groups were also found in relation to awareness and learning outcomes. In general, more women in the postpartum follow-up group reported how the information in the program had created awareness and self-reflection and contained important reminders that facilitated attachment and bonding with the baby. These findings are in line with previous research showing that many noncompleters also experience the clinical benefit of programs [53] and that the greatest benefits of interventions can be observed among those with a completion rate in the top quartile [28]. However, the postpartum follow-up group dropped out later and thus may have found the program more beneficial or positive in general. From a prevention and treatment perspective, these findings are important because the intervention was designed to prevent the onset or worsening of perinatal depression among pregnant and postpartum women and, thus, negative consequences for the child as well [6,54]. Our findings of different learning and awareness experiences in the 2 groups could be contributed to both individual differences between the participants and factors related to the intervention. However, there were no differences between participants in the groups in terms of marital status, education, or employment. Participants in both groups also had few symptoms of depression, and there were no differences in quality of life or negative affect scores between the participants before they started the intervention.

Opportunities for improving the program were mentioned by both groups. Although women in the pregnancy group typically suggested that the content of the program should be better adapted to the target group, women in the postpartum follow-up group emphasized that they wanted more flexibility in the program. Although participants in internet-delivered interventions usually can choose where and when they want to work with the program, they requested more flexibility and suggested links to more information on some subjects. This is in line with previous research, showing that inflexibility is a common experience among participants in internet-delivered treatment that can lead to nonadherence [55].

Some important limitations should be noted when interpreting the results of this study. First, the women who participated were typically living with a partner, well educated, and employed. They also scored low on depressive symptoms and high on subjective well-being. Thus, a limitation of this study is the potential of sampling bias. Maternal mental health can change during pregnancy and the postpartum period, and therefore, universal health programs must reach as many women as possible, including those who are doing well. However, future research should include a more diverse sample of women, for example, women with lower socioeconomic status, a history of depressive disorders, or current mild to moderate symptoms of depression (ie, Edinburgh Postnatal Depression Scale score≥10). Despite these limitations, the result of this study adds to the literature on user experiences with dropout from a universal internet-delivered program in the perinatal period.

### Conclusion

The results of this study showed that there were differences between women who dropped out of the intervention during pregnancy and the postpartum follow-up phase. In general, more women in the postpartum follow-up group reported that the program was user-friendly. They became more conscious of their own thoughts and feelings and perceived that the program had provided them with more new knowledge and practical information than participants in the pregnancy group. However, women in both groups suggested several opportunities to improve the program. Although women in the pregnancy group typically suggested that the content of the program should be better adapted to the target group, it was in general more pronounced in the postpartum follow-up group that they wanted more flexibility in the program. The reported differences between groups should be further examined.

## Abbreviations

COREQ: Consolidated Criteria for Reporting Qualitative Research
SWOT: strengths, weaknesses, opportunities, and threats
